# Supplementary material for: P113 is a merozoite surface protein that binds the N terminus of Plasmodium falciparum RH5
Source: Nat Commun. 2017 Feb 10;8:14333. doi: 10.1038/ncomms14333 (PMC5309799; doi:10.1038/ncomms14333)
Supplement: Supplementary Information — Supplementary Figures and Supplementary Table. [file ncomms14333-s1.pdf]

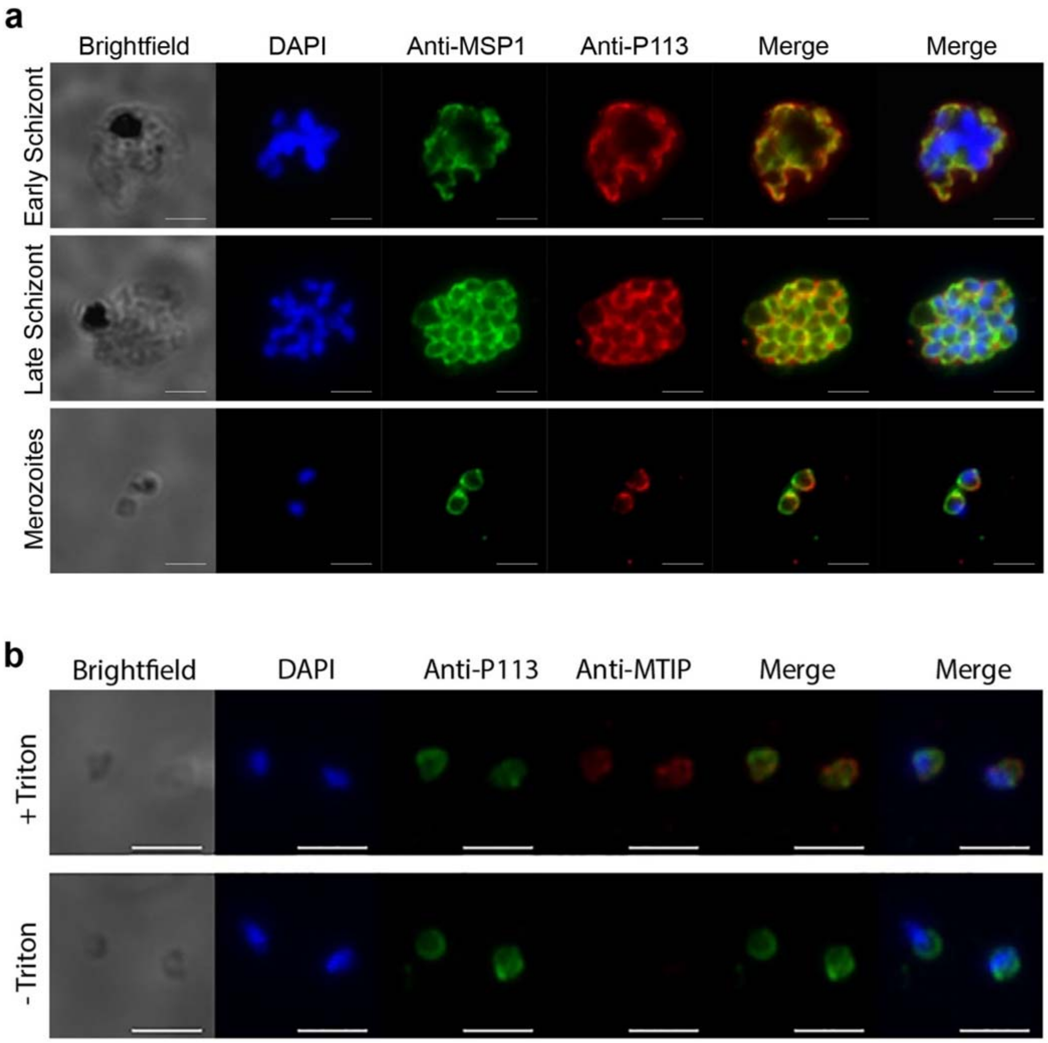

Supplementary Figure 1. P113 is expressed in both early and late-stage *P. falciparum* schizonts and on the merozoite surface. Fixed blood stage *P. falciparum* schizonts and free merozoites were co-stained with antibodies raised to P113 and the merozoite surface marker anti-MSP1 (**a**), or an inner membrane complex marker, MTIP (**b**) as indicated. In (**b**), fixed merozoites were stained either with (+) or without (-) a membrane permeabilisation step using the non-ionic detergent Triton X-100. Antibodies to P113 - but not the inner membrane marker MTIP - stained both Triton X-100 treated and untreated samples demonstrating that P113 is located on the surface of the merozoite. P113 staining often - although not always - exhibited an asymmetric distribution on the merozoite surface. Nucleic acid is stained with DAPI and scale bars represent 3μm.

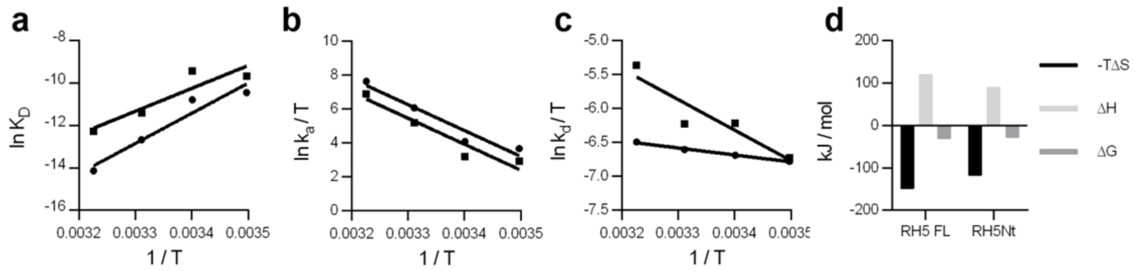

Supplementary Figure 2. Thermodynamic surface plasmon resonance analysis of P113 interaction with RH5 FL and RH5Nt. P113 Y1-N653 was injected over immobilised RH5FL (500 RU) and RH5Nt (240 RU) (150RU of Cd4 control was immobilised in the reference flow cell) at different temperatures (13-37˚C) and concentrations (2 fold serial dilution of 2 to 0.0625µM). Kinetic association and dissociation values were calculated. From these were plotted a van't Hoff plot (a) and Eyring plots (b and c) from which the entropy, enthalpy and free energy components of the interactions could be calculated (d). Circles represent RH5FL and squares RH5Nt.

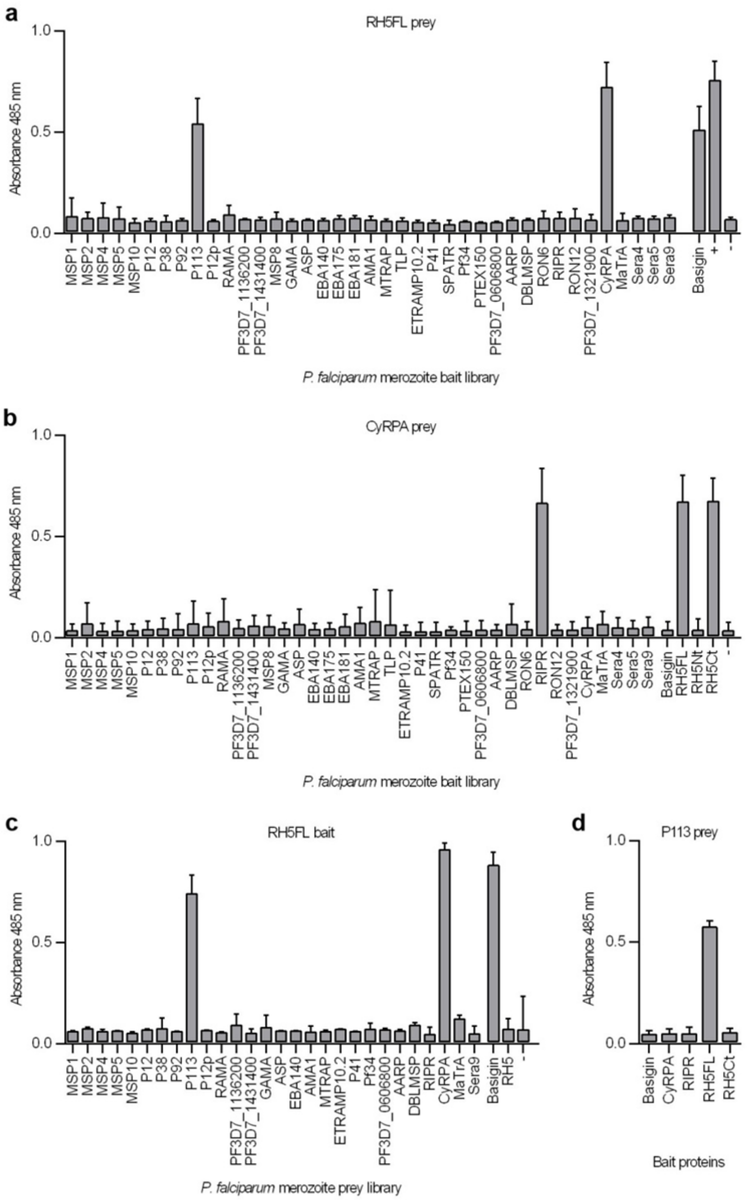

Supplementary Figure 3. Systematic screening for direct interactions between *Plasmodium falciparum* merozoite proteins within the RH5 complex using AVEXIS. The indicated *P. falciparum* merozoite proteins were expressed as either pentameric, β-lactamase-tagged preys or monomeric biotinylated baits and systematically tested for direct interactions using the AVEXIS assay. (**a**) RH5FL interacts with P113 and CyRPA. (**b**) CyRPA interacts with RIPR, RH5FL and RH5Ct. (**c**) RH5FL bait was screened against a merozoite protein library presented as preys. Direct interactions with P113, CyRPA and basigin were observed. (**d**) P113 prey did not interact with CyRPA, RIPR or basigin. Bars represent means ± 95% confidence intervals; $n = 3$, a representative of at least two independent experiments is shown; where indicated, controls were the rat Cd200R prey presented to Cd200 bait (+ve) or Cd4d3+4 tag alone (-ve).

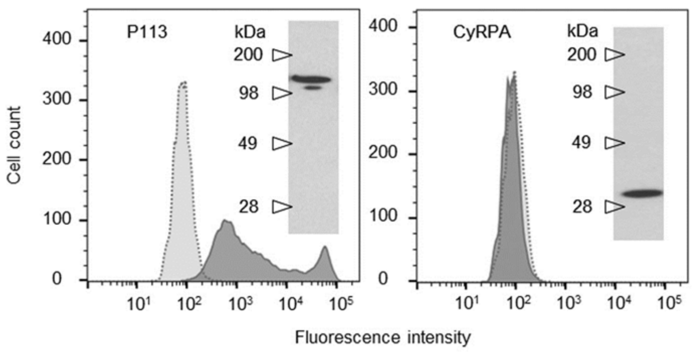

Supplementary Figure 4. P113, but not CyRPA, is tethered to the plasma membrane when ectopically expressed in HEK293 cells. The entire endogenous sequences for P113 and CyRPA (including signal peptides and predicted GPI-signal sequences) were expressed for 24h in HEK293 cells and cell surface expression was assessed by staining unfixed and unpermeabilized cells with anti-P113 or anti-CyRPA antibodies respectively. Histograms show cells transfected with P113 (left panel) or CyRPA (right panel) and stained with the corresponding antibody (dark gray) relative to negative control mock transfections (light gray). Insets show Western blots of culture supernatants 48h after transfection. Both P113 and CyRPA were detected at their expected masses.

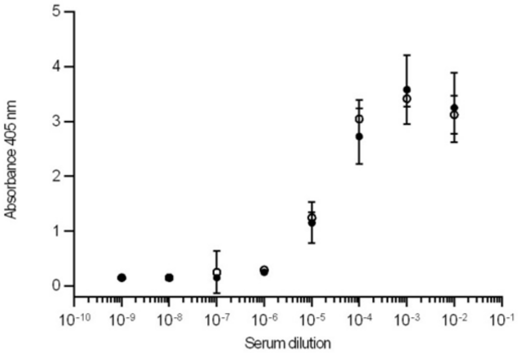

Supplementary Figure 5. An amph-vaccine made from a chemically synthesized peptide based on RH5Nt elicited good antibody titres. The antibody responses in sera from two rabbits (open and closed circles) immunised with the amph-vaccine based on RH5Nt were quantified by ELISA against recombinant, biotinylated RH5Nt immobilised on a streptavidin-coated microtitre plate. Data points present means ± 95% confidence intervals; $n = 3$.

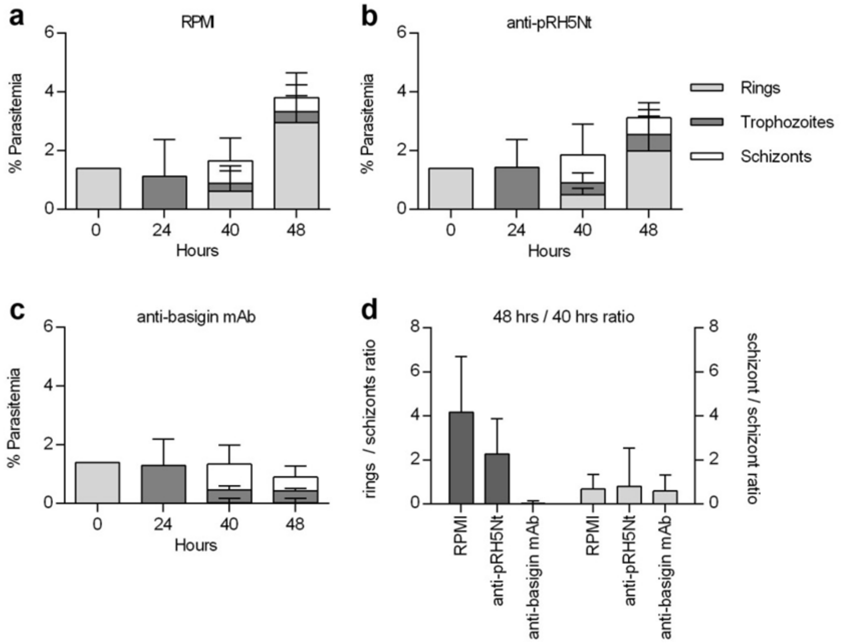

Supplementary Figure 6. Anti-RH5Nt antibodies inhibit parasite growth by preventing invasion. A time course of blood stage development was performed in the presence of polyclonal antibodies against RH5Nt (pRH5Nt), media alone or an anti-basigin mAb. Smears were made at intervals over 48 hrs from blood stage cultures of *P. falciparum* 3D7 in RPMI alone (a), with polyclonal antibodies against pRH5Nt in RPMI at 4 mg/ml (b) or basigin monoclonal antibodies in RPMI at 10 µg/ml (c). The number of rings, trophozoites and schizonts at each time point (0, 24, 40 and 48 hours) were counted after being smeared, fixed and stained with Giemsa and 2000 erythrocytes were examined by light microscopy. (d) The ratio of rings at 48 hrs to schizonts at 40 hrs is shown in dark grey and the ratio of schizonts at 48 and 40 hrs is shown in light grey. The bars represent means ($n = 3$) and errors bars 95% confidence intervals.

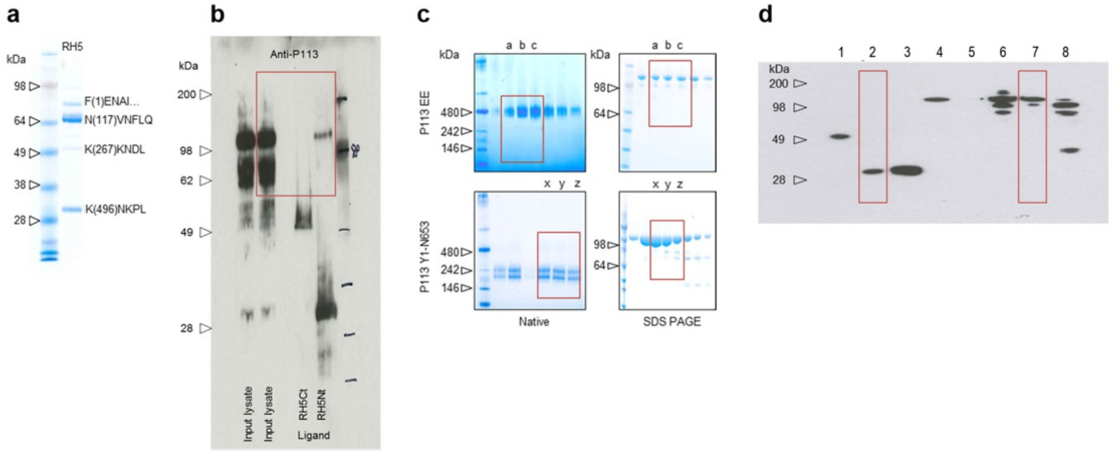

Supplementary Figure 7. Uncropped gels and blots used to produce figures. Figure showing images of uncropped gels used in figures: (**a**) Fig. 1a. (**b**) Fig. 2c. (**c**) Fig. 4e. (**d**) and Supplementary Fig. 4; red boxes indicate the approximate cropped regions used to produce the figures. In (**b**), the bands detected with the anti-P113 antiserum at ~50kDa and ~30kDa correspond to the RH5Ct and RH5Nt proteins leached from the streptavidin-coated beads during the elution step of the biochemical purifications. These proteins are detected with the anti-P113 antiserum because the P113 protein used as the immunogen and the RH5Ct and RH5Nt proteins immobilised on the beads all contain a common protein tag: rat Cd4 domains 3 and 4. In (**d**), the different lanes represent supernatants from transfected cells detected by Western blotting using pooled antibodies to CyRPA and P113: (1) CyRPA-Cd4 recombinant protein at 48 hrs (positive control); untagged (endogenous sequence) of CyRPA at 48 hrs or 6 days (lanes (2) and (3) respectively); P113-Cd4 recombinant protein (positive control) at 24 hrs or 48 hrs (lanes (4) and (6) respectively); untagged (endogenous sequence) of P113 at 24 hrs, 48 hrs and 6 days (lanes (5), (7) and (8), respectively).

Exp. No.	Analyte	Ligand	k_a (10^5 M^{-1} s^{-1})	k_d (s^{-1})	$K_{D\ calc.}$ (μM)	$t_{1/2}$ (s)
1	RH5Ct	basigin	3.42 ±0.01	0.2926 ±0.0002	0.9	2.5
2	RH5FL	basigin	1.82 ±0.02	0.244 ±0.001	1.3	2.8
3	RH5Nt	P113	3.0 ±0.1	0.793 ±0.009	2.7	0.9
4	RH5FL	P113	7.8 ±0.2	0.231 ±0.003	0.3	3.2
5	RH5FL	P113 Y1-N653	7.53 ±0.04	0.412 ±0.002	0.5	1.9
6	RH5FL	P113 Y1-K197	2.84 ±0.05	0.307 ±0.002	1.1	2.3
7	P113 Y1-N653	RH5Nt	3.04 ±0.09	1.54 ±0.03	5.0	0.5
8	P113 Y1-N653	Synthetic RH5Nt	2.16 ±0.06	1.24 ±0.01	5.7	0.6

Supplementary Table 1: A table summarising the biophysical binding parameters for RH5 interactions measured using surface plasmon resonance in this study. All values were determined by fitting the whole binding data (association and dissociation phases) to a simple (1:1) binding model using gel filtrated monomeric proteins as analytes, and biotinylated monomeric ligands immobilised on a streptavidin-coated sensor chip. Values for association (k_a) and dissociation (k_d) rate constants are means ± SE taken from a representative experiment. All experiments were performed at least twice using independent protein preparations except experiments 6 and 8 which were performed once; at least six serial dilutions of the analyte were used in every experiment. K_{Dcalc} was calculated from the association and dissociation rate constants ($K_{Dcalc} = k_d/k_a$) and provides an independent measure of the K_D determined from equilibrium binding analysis; interaction half-lives ($t_{1/2}$) = ln2/k_d.
